# Identifying Time Measurement Tampering in the Traversal Time and Hop Count Analysis (TTHCA) Wormhole Detection Algorithm

**DOI:** 10.3390/s130506651

**Published:** 2013-05-17

**Authors:** Jonny Karlsson, Laurence S. Dooley, Göran Pulkkis

**Affiliations:** 1 Department of Communication and Systems, The Open University, Walton Hall, Milton Keynes, MK7 6AA, UK; E-Mail: laurence.dooley@open.ac.uk; 2 Department of Business, Information Technology and Media, Arcada University of Applied Sciences, Jan-Magnus Janssons plats 1, Helsinki 00550, Finland; E-Mail: goran.pulkkis@arcada.fi

**Keywords:** mobile networks, MANET, MANET security, routing security, wormhole attack, hop count, queuing delay, packet processing time, TTHCA, MHA

## Abstract

*Traversal time and hop count analysis* (TTHCA) is a recent wormhole detection algorithm for *mobile ad hoc networks* (MANET) which provides enhanced detection performance against all wormhole attack variants and network types. TTHCA involves each node measuring the processing time of routing packets during the route discovery process and then delivering the measurements to the source node. In a *participation mode* (PM) wormhole where malicious nodes appear in the routing tables as legitimate nodes, the time measurements can potentially be altered so preventing TTHCA from successfully detecting the wormhole. This paper analyses the prevailing conditions for time tampering attacks to succeed for PM wormholes, before introducing an extension to the TTHCA detection algorithm called Δ*T Vector* which is designed to identify time tampering, while preserving low false positive rates. Simulation results confirm that the Δ*T Vector* extension is able to effectively detect time tampering attacks, thereby providing an important security enhancement to the TTHCA algorithm.

## 1. Introduction

A *Mobile ad hoc Network* (MANET) is a self-configuring arrangement of wireless nodes which can communicate with each other without requiring core infrastructure such as routers and base stations. They can be deployed in a range of application domains including military communications, vehicular and sensor networks, and as an access mechanism to the Internet in scenarios where nodes are out-of-radio range, such as in underground transport systems.

The open nature and absence of dedicated routers mean that MANETs are especially vulnerable to routing attacks [1,2] which can lead to severe disruption of network communications. The *wormhole attack* [3] is one of the most serious MANET routing threats since it is relatively easy to launch, difficult to detect and can yet cause significant communications disruption. A wormhole creates a fictive shortcut link in the network with the intention to attract data packets to traverse specific nodes. It involves two collaborating malicious nodes forwarding routing packets to each other. When a malicious node captures a routing packet, it is encapsulated within a new packet and tunnelled to the other wormhole node, which then extracts the routing packet before relaying it to its neighbours. As a consequence, malicious nodes can appear as neighbours despite being located several hops from each other.

Wormhole attacks can be launched in two ways: *hidden mode* (HM) and *participation mode* (PM) [4]. The former captures and forwards routing packets to each other without modifying the actual packets, so the wormhole nodes never appear in routing tables. In contrast, PM nodes process routing packets as any pair of legitimate nodes and thus appear in a wormhole infected route as two contiguous nodes.

Wormhole nodes can forward routing packets to each other using either an *in-band* (I-B) or *out-of-band* (O-B) communication link. I-B tunnels packets between the malicious nodes via genuine network nodes so it is easy to launch, while the O-B link is more complex because it requires an external communication channel, *i.e.*, network cable or directional antenna, to establish a direct link between the wormhole nodes.

Designing effective and robust wormhole detection schemes means considering all four modes with each mandating different requirements upon the detection mechanism. Various detection strategies have been proposed and these can be broadly classified into: (*i*) neighbour validation and (*ii*) end-to-end techniques.

Neighbour validation schemes like packet leashes [3] and [5] are only effective for HM wormhole attacks because they rely on every node checking the validity of its neighbours and since PM wormhole nodes appear as legitimate neighbours in a route, they can avoid being detected by simply ignoring the validity check. Other schemes like *statistical wormhole apprehension using neighbours* (SWAN) [6] identify a wormhole by the number of neighbours, though this is only effective for HM wormholes since PM wormholes do not increase the number of neighbours for a legitimate node.

In contrast, end-to-end detection techniques measure and analyse node activity and route features such as the geographical positions of nodes [7–11], the frequency of node appearances in routes [9–11], *hop count* (HC) information [12] or *round trip time* (RTT) of routing packets [13–16]. Such techniques are typically used to detect PM wormholes, but have a number of recurring limitations including, the inability to detect all wormhole variants, the requirement of dedicated hardware, reliance on certain MANET environments, and high computational overheads and/or bandwidth loads upon the network.

The *traversal time and hop count analysis* (TTHCA) algorithm is a new wormhole detection technique [17] designed as a security extension to the *ad hoc on demand distance vector* (AODV) [18] routing protocol. It combines the benefits of RTT-based approaches with HC analysis, to provide improved detection for all wormhole types, under a variety of network scenarios. RTT-based wormhole detection schemes, such as *wormhole attack prevention* (WAP) [13], *transmission time-based mechanism* (TTM) [15] and *delay per hop indication* (DelPHI) [14], offer low overhead solutions in terms of hardware, computation and throughput, but have the limitation that variations in a node's packet processing time *i.e.*, the sum of the queuing delay and service time must be small. In a real MANET, nodes can exhibit high packet processing time variations, a feature the *neighbour probe acknowledge* (NPA) method [16] addresses by employing the standard deviation of the RTT as an accurate metric. NPA has not however, been tested in large scale networks and is inherently computationally heavier than either TTHCA or other RTT-based techniques because it uses encryption and time-stamped digital signatures to guarantee the security of the routing packets. In TTHCA, *packet traversal times* (PTT) are measured instead of the RTT of a routing packet, as this more accurately reflects the distance between a source and destination node. The corollary is that TTHCA affords significantly superior wormhole detection and lower *false positive* (FP) performance than RTT-based solutions, while concomitantly affording low computational overheads.

A potential drawback of TTHCA is that under specific conditions, PM wormhole nodes can alter the time measurements and prevent the wormhole from being detected. In TTHCA, PTT is estimated by initially allowing each intermediate node to measure the packet processing time of the AODV *route request* (RREQ) and *route reply* (RREP) packets, before adding this measurement value Δ*T_i_* to a Δ*T_TOT_* parameter in the RREP packet. Upon receiving the RREP, the source node can calculate PTT by subtracting Δ*T_TOT_* from the RTT. A wormhole is suspected if the PTT is unrealistically high in relation to the HC. By falsely increasing Δ*T_TOT_*, a PM wormhole node can evade being detected because this results in a smaller PTT than is in fact, the case. Time tampering attacks are not relevant to HM wormholes because as mentioned above, they never process the routing packets.

This paper analyses the time tampering problem and investigates its impacts on TTHCA wormhole detection performance. A solution is presented to accurately identify time tampering in PM I-B wormholes by introducing a Δ*T Vector* extension into the TTHCA algorithm. The Δ*T Vector* replaces the Δ*T_TOT_* parameter in the RREP packet with a list of the individual Δ*T_i_* values from all intermediate nodes. A malicious node must thus produce a falsely inflated Δ*T_i_* in order to perform a successful time tampering attack. By using the Δ*T Vector* extension, a tampered Δ*T_i_* can be accurately identified by the source node as it typically is significantly higher than a healthy Δ*T_i_*.

The remainder of the paper is organized as follows: Section 2 presents a brief overview of the TTHCA algorithm before Section 3 investigates time tampering attacks and the specific conditions necessary for this security breach to ensue. The new Δ*T Vector* extension is then introduced in Section 4 and its performance analysed in Section 5 for diverse MANET scenarios. Finally, some concluding comments are provided in Section 6.

## 2. The Traversal Time and Hop Count Analysis (TTHCA) Algorithm

In TTHCA, a source node firstly measures the RTT of the AODV route discovery packets, which is the time between sending the RREQ packet and receiving the RREP packet. Each intermediate node measures the processing time of the RREQ and RREP packets *(*Δ*T_i_*) and this is added to the Δ*T_TOT_* parameter in the RREP packet. Hence, once a RREP packet is received by the source node:

(1) $$\Delta T_{\text{TOT}}=\sum_{i=1}^{\text{HC}}\Delta T_{i}$$

and the PTT is calculated from:

(2) $$\text{PTT}=\frac{\text{RTT}-\Delta T_{\text{TOT}}}{2}$$

A wormhole is then suspected if:

(3) $$\frac{\text{PTT}}{\text{HC}}>\frac{R}{S}$$

where *R* and *S* are respectively the maximum radio range per node and the propagation speed (*i.e.*, 3 × 10^8^ m/s).

When a wormhole is suspected, all intermediate nodes on the route are added to a *graylist* [12] which is broadcasted throughout the MANET together with a new RREQ. All graylist nodes are then omitted during the next route discovery procedure resulting in a new unique route. Graylist broadcasting is repeated until a healthy route is found.

## 3. Time Tampering in TTHCA

The TTHCA wormhole detection algorithm is predicated on the assumption that a wormhole route will exhibit an unrealistically high PTT per HC. Wormhole nodes however, can potentially prevent TTHCA from detecting infected routes by adding a fictive packet processing time Δ*T_F_* to the Δ*T_TOT_* parameter of the RREP packet. It is important to stress that time tampering is not a modification attack *per se* as the PM wormhole node never alters any routing packet parameters, but instead produces false measurement information. This means schemes designed to prevent packet alteration by for example, encrypting all routing packet parameters, will be ineffectual against a TTHCA time tampering attack.

As a wormhole infected route has a high PTT/HC, the malicious nodes must artificially produce a lower PTT than in reality for that route to avoid detection and this can be accomplished by increasing Δ*T_TOT_*. Since Δ*T_TOT_* ≫ *PTT* and Δ*T_i_* may incur large fluctuations due to for example, variable network traffic loads, it is difficult for the wormhole nodes to be aware of exactly how to set Δ*T_F_* as it must be precisely defined within the narrow time window that exists to effectively achieve time measurement tampering. This window is bounded by:

(4) $$\left(\text{RTT}-\Delta {\text{T}}_{\text{TOT}}-2\text{HC}\;\frac{R}{S})\leq \Delta T_{F}\leq (\text{RTT}-\Delta {\text{T}}_{\text{TOT}}\right)$$

So if the tampered Δ*T_F_* is too small, TTHCA is still able to detect the route as a wormhole because PTT/HC is higher than the threshold in Equation (3). Conversely, if Δ*T_F_* is made too high the resulting PTT at the source node will be negative.

Pragmatically it is not feasible for a malicious node to exactly know the time tampering window since it can only be aware of the values of *R* and *S* in Equation (4). Successful time tampering is still feasible however, if the malicious nodes (M_1_ and M_2_) can estimate the RTT of the wormhole link (*RTT_WH_*). In an I-B link, *RTT_WH_* can have high variations due to variable packet processing times at the nodes through which the wormhole is tunnelled, making the precise estimation of *RTT_WH_* challenging. One approach for estimating *RTT_WH_* for PM wormhole links is to use tightly synchronized clocks. During route discovery, wormhole node M_1_ adds exact time information as an adjunct parameter within the tunnelled packet as it forwards the RREQ to the other malicious node M_2_. Upon receiving this tunnelled RREQ, M_2_ estimates the precise propagation delay of the RREQ through the wormhole *t_RREQ_* by comparing the received time information with its own clock. A similar process occurs when M_2_ returns RREP to M_1_, with time information again being added as the RREP is tunnelled to M_2_. When M_1_ receives the tunnelled RREP, it calculates *t_RREP_* to give:

(5) $${\text{RTT}}_{\text{WH}}\;=\;t_{\text{RREQ}}\;+\;t_{\text{RREP}}$$

M_1_ then adds the fictive time value Δ*T_F_* defined as:

(6) $$\Delta T_{F}={\text{RTT}}_{\text{WH}}\;-\;2\frac{R}{S}$$

to Δ*T_TOT_* of the RREP in addition to its own Δ*T_i_*.

Alternatively, the wormhole nodes can split the time tampering attack into two steps. Firstly, M_2_ adds the fictive value:

(7) $$\Delta T_{F1}=t_{\text{RREQ}}-\frac{R}{S}$$

before M_1_ adds:

(8) $$\Delta T_{F2}=t_{\text{RREP}}-\frac{R}{S}$$

So Δ*T_F_*= Δ*T_F1_+* Δ*T_F2_* is then added to Δ*T_TOT_*.

**Figure 1.**
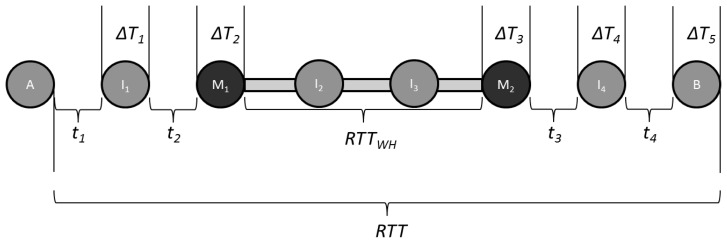
MANET scenario where A and B are the source and destination nodes, M_1_ and M_2_ are malicious wormhole nodes, *t_i_* is *2 x PTT* between two successive nodes, Δ*T_i_* is the routing packet processing time, RTT is the round trip time of the route, and *RTT_WH_* is the RTT of the wormhole link.

To illustrate the conditions that must exist for TTHCA time tampering to be achieved, consider the MANET example in Figure 1, where a PM I-B wormhole is formed by nodes M_1_ and M_2_ which tunnel routing packets between each other via I_2_ and I_3_.

It is assumed for simplicity that all nodes are in an idle state, have identical hardware and the inter-node distance is the same, so the *t_i_* and Δ*T_j_* values are constant. Let *t_i_*= 1,600 ns for all *i* and Δ*T_j_*= 8 ms for all *j*, where *j = i + 1*. If *RTT_WH_*= 16.0048 ms then *RTT *= 56.0112 ms. For this PM I-B scenario, the HC is 5 and Δ*T_TOT_*= 40 ms, so from Equation (2), source node A calculates *PTT *= 8.0056 ms giving *PTT/HC *= 1.60112 ms. If it is assumed *R *= 250 m, then from Equation (3) the upper bound for *PTT/HC *= 833 ns which means TTHCA will successfully detect the wormhole. Using Equation (4), it can be determined that both I_2_ and I_5_ are able to prevent detection by increasing Δ*T_TOT_* within the range:

$$16.002867\;\text{ms}\leq \Delta T_{F\;}\leq \;16.011200\;\text{ms}$$

This means the time tampering window is only 8.33 μs wide and while this is a stringent constraint, if synchronized clocks are being used by both M_1_ and M_2_, it is still realistically an achievable design tolerance.

Analysis for a wide range of network and wormhole attack conditions reveals that a sufficient and necessary condition for a wormhole to avoid being detected is to uphold either Equations (6) or (7) and (8). In this PM I-B example, both M_1_ and M_2_ will calculate Δ*T_F_*= 16.003133 ms which implies the tampered value falls within the window Equation (4) to avoid being discovered. In these circumstances, the false measurement Δ*T_TOT_*= 56.003133 ms so from Equation (2), the source node A measures *PTT *= 4,033 ns and *PTT/HC *= 806 ns meaning this wormhole route will go undetected by TTHCA.

## 4. Δ*T Vector* TTHCA Extension

Section 3 showed that the essential condition for the TTHCA algorithm to be unable to detect a wormhole route is for the malicious nodes to increase Δ*T_TOT_* within the strict bounds defined in Equation (4). Any successful tampered Δ*T_TOT_* will always be greater than the actual Δ*T_TOT_* though simply analysing Δ*T_TOT_* as a sum of individual Δ*T_i_* values will not necessarily identify the wormhole route because these usually exhibit high variance.

**Figure 2.**
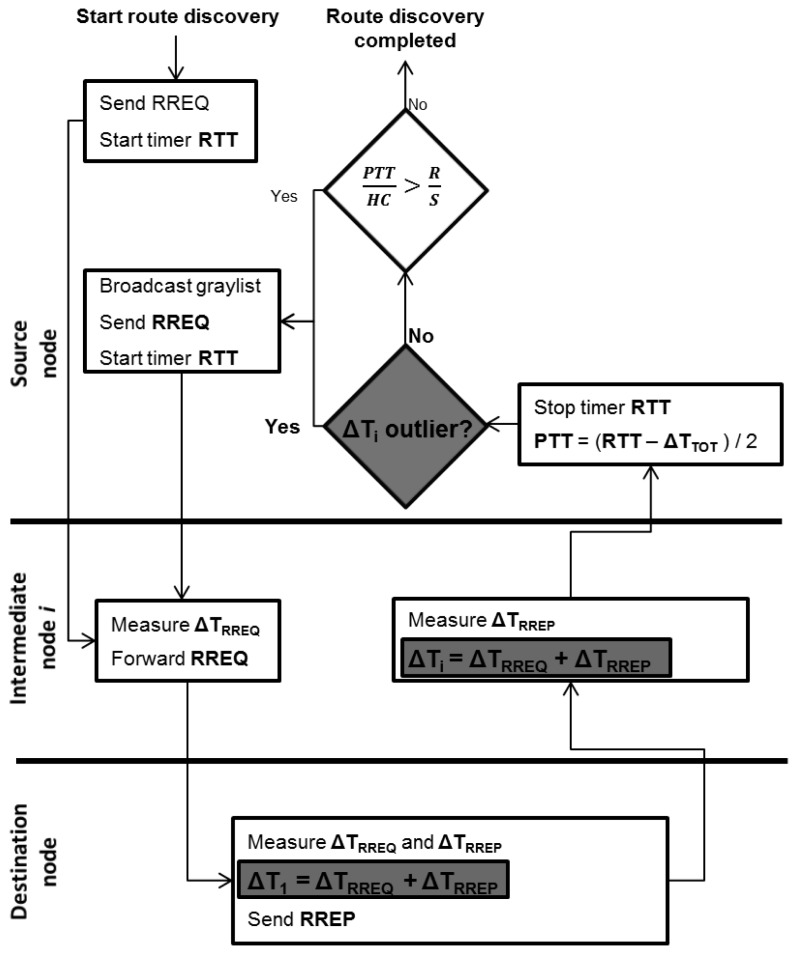
TTHCA route discovery with the ΔT Vector extension (RTT= round trip time, RREQ= route request, RREP = route reply, ΔT = packet processing time, *PTT* = packet traversal time, *HC* = hop count, *R* = radio range, *S* = propagation speed).

In this paper, to analyse Δ*T_i_* for each intermediate node, Δ*T_TOT_* is replaced by a new Δ*T Vector* comprising all the measured Δ*T_i_* values. This extension means that some new features for the TTHCA route discovery process are introduced to support the embedding of the Δ*T Vector* as shown in the Figure 2 flowchart, with the shaded blocks highlighting these new elements.

The RREQ and *graylist* broadcast procedures remain as in original TTHCA [17], but instead of using a Δ*T_TOT_* parameter, the Δ*T Vector* is included in the RREP packet by the destination node. The time taken from receiving the RREQ until sending the RREP at the destination node is added as the first element Δ*T_1_*. Each intermediate node receiving and forwarding the RREP then adds its Δ*T_i_* (ΔT_RREQ_ + ΔT_RREP_) as a new element in the Δ*T Vector*.

When the RREP is received by the source node, each Δ*T_i_* element of the Δ*T Vector* consists of the processing times incurred by the RREQ and RREP packets. If a PM wormhole attack is launched alongside a time tampering attack, at least one of the Δ*T Vector* elements will be falsely increased in accordance with Equations (6), (7) and (8). A suitable outlier detection technique can then be applied to identify tampered Δ*T_i_* values (see Section 4.1) from the Δ*T Vector* dataset. If a suspicious Δ*T_i_* is identified, TTHCA then requests a new route by issuing a *graylist* broadcast. If no suspicious Δ*T_i_* is found, the normal PTT/HC analysis is performed for both HM and PM wormhole detection.

### 4.1. Identifying Tampered ΔT_i_ Measurement

The Δ*T Vector* extension is founded on the premise a malicious node can only modify its own Δ*T_i_* which is a pragmatic assumption since in real MANET environments routing packets must be secured from modification attacks for the routing process to be trustworthy. A wormhole link typically consists of two malicious nodes, so a Δ*T Vector* received through any wormhole infected route will include either one or two tampered Δ*T_i_* values. It is possible to distinguish tampered Δ*T_i_* values from healthy Δ*T_i_* measurements by applying an appropriate outlier detection technique, such as the Grubb's test [19], Dixon's Q-test [20] or the Box plot method [21], though several conditions can affect the performance of the chosen outlier method. In this context, two distinct MANET scenarios are defined:

CASE 1: A node has been a part of the network for some time and generated a track record of Δ*T_i_* values gained from Δ*T Vectors* from earlier route discovery procedures. In this scenario, the availability of a large number of Δ*T_i_* samples can be reasonably assumed.
CASE 2: A node has joined the MANET for the first time and so the only available Δ*T_i_* values are those existing in the Δ*T Vector*.

Due to the inherently dynamic nature of a MANET, several different types of Δ*T_i_* distributions can arise which will impact on the performance of the outlier detection scheme. The ideal is when all MANET nodes have identical hardware and the network traffic loads are low. Such a condition would result in negligible Δ*T_i_* variations and time tampering is then straightforward to detect. This is not however, a realistic MANET situation because there are a myriad of factors which can cause Δ*T_i_* variations. For example, mixed node processing capacities and packet service times, allied with high network traffic loads in certain parts of the MANET can lead to queuing delays at specific nodes.

In a heterogeneous MANET consisting of uniformly distributed nodes where the network traffic load is low and there are no queuing delays, the Δ*T_i_* values can be assumed to follow a linear distribution. In MANETs with high network traffic load variations however, some of the Δ*T_i_* values will include queuing delays which will be much greater than the actual packet service times [22]. The Δ*T_i_* values will then tend to follow a nonlinear distribution where a small portion of the Δ*T_i_* values are significantly higher than the average. For such a distribution, it is very challenging to discriminate a tampered from a normal Δ*T_i_* value as a modified Δ*T_i_* can potentially be lower than a healthy Δ*T_i_* if the tampered measurement contains no queuing delay, while the healthy Δ*T_i_* does.

The outlier detection method selected for time tampering detection purposes must therefore be applicable to both large and small Δ*T_i_* datasets *i.e.*, CASE 1 and CASE 2 respectively, as well as for both linearly and non-linearly distributed measurements.

## 5. Performance Analysis

The performance of the Δ*T Vector* extension has been rigorously analysed using the Dixon Q-test [20] as the outlier detection technique to identify tampered Δ*T_i_* values for a PM I-B wormhole infected route. The Q-test was chosen because of its simplicity and applicability to small and large datasets, making it appropriate for both the CASE 1 and CASE 2 scenarios. While the Q-test is only capable *per se* of detecting a single outlier, it can be applied to detect either one or two tampered Δ*T*_i_ values provided the right-tailed variant is used to separately test the two largest Δ*T*_i_ values. The outlier test is thus performed by first ranking the Δ*T vector* in order and then respectively calculating two Q values:

(9) $$Q_{1}=\frac{\Delta {\text{T}}_{\text{HC}}\;-\;\Delta {\text{T}}_{\text{HC}-1}}{\Delta {\text{T}}_{\text{HC}}\;-\;\Delta {\text{T}}_{1}}$$

(10) $$Q_{2}=\frac{\Delta {\text{T}}_{\text{HC}-1}\;-\;\Delta {\text{T}}_{\text{HC}-2}}{\Delta {\text{T}}_{\text{HC}-1}\;-\;\Delta {\text{T}}_{1}}$$

Time tampering is suspected if either *Q_1_* or *Q_2_* is greater than the corresponding critical *Q*-value for the chosen confidence level. For this analysis, a low confidence level (80%) has been chosen, since from a security perspective, a higher time tampering detection rate is preferable to a low FP detection.

Both the time tampering and FP detection performance for the Δ*T Vector* extension were analysed using a custom designed tool which simulated differently sized Δ*T Vectors* to reflect variable HC routes. Δ*T_i_* values were produced by randomly generating packet processing times for each node, with variable inter-node distances considered for each route.

The *operating system* (OS) for each MANET node was assumed to support multiprogramming with a scheduler assigning equal time slices to each process in rotation. Such an OS approximately implements processor-sharing so a logical processor executes each multiprogrammed task, with the processing capacity of a logical processor being the ratio of the physical processor capacity and the multiprogramming level. While nodes will typically have different physical processing capacities and multiprogramming levels, the equivalent multiprogramming level for each node will be relatively stable. A MANET having logical processors with diverse, yet stable processing capabilities is thus assumed to handle routing packets, so the corresponding packet service times for each node is assumed to be constant. Many concurrent route detection procedures can lead to routing packet queues in MANET nodes, since received routing packets must be sequentially processed to uphold route table updating requirements. For this reason, the packet processing times (Δ*T_RREQ/RREP_*) have been generated using the M/D/1 queuing model [23], which assumes Poisson-distributed packet arrivals, deterministic service times of routing packets, a single central processing unit and an infinite maximum queue length. Hence, at each node:

(11) $$\Delta T_{\text{RREQ}/\text{RREP}}\;=\;\text{queuing}\;\text{delay}\;+\;T_{S}\;=\;\frac{T_{S}\;(2-\rho )}{2\;(1-\rho )}$$

where *T_S_* and *ρ* are the routing packet service time and network traffic load upon a node respectively. Variations in both node processing capacity and multiprogramming level are reflected by using random *T_S_* values from a linear probability distribution of different intervals denoted by the *relative standard deviation* (*σ_R_*), which is the standard deviation of all the packet service times divided by their average. Variable network traffic loads between nodes are mirrored by randomly selecting *ρ* on each node within the interval *0* ≤ *ρ* ≤ *ρ_max_*, where *ρ_max_* is the maximum network traffic load per node.

Time tampering detection performance for the CASE 1 and CASE 2 scenarios will now be respectively considered, where time tampering attacks on TTHCA are simulated in accordance with Equations (7) and (8). Note that the results presented relate solely to the Δ*T Vector* time tampering detection performance of the TTHCA algorithm, and not to the wormhole attack detection rates, which have already been rigorously presented in [17]. The simulation parameter settings used throughout the experiments are given in Table 1, with a detailed description of the customised simulation tool being provided in Appendix A.

### 5.1. CASE 1: MANET Nodes with ΔT_i_ Track Records

**Figure 3.**
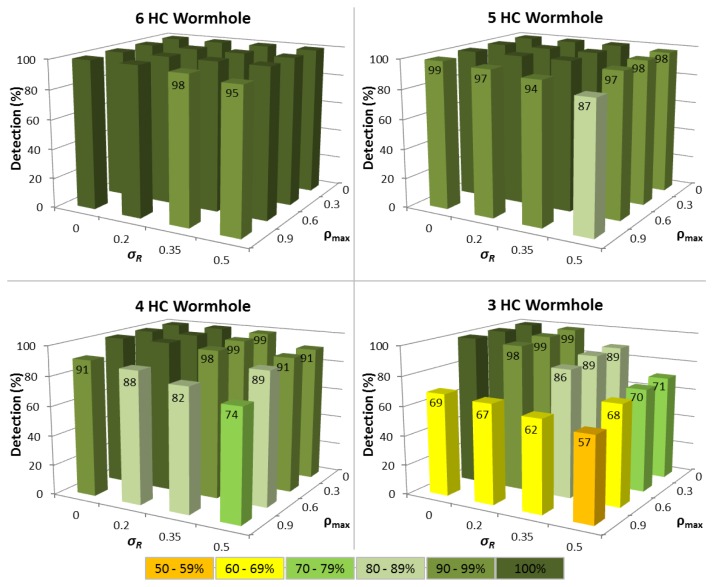
Time tampering detection performance for different wormhole HC for variable network traffic loads (*ρ_max_*) and routing packet service times (*σ_R_*) with at least 15 Δ*T_i_* samples available.

In the first series of experiments, the situation where a node has been in the MANET for a period of time is analysed and there are at least 15 Δ*T_i_* values available. Figure 3 shows the impact of variations in both routing packet service time (*σ_R_*) and network traffic load (*ρ_max_*) upon the time tampering detection performance for different wormhole lengths.

**Figure 4.**
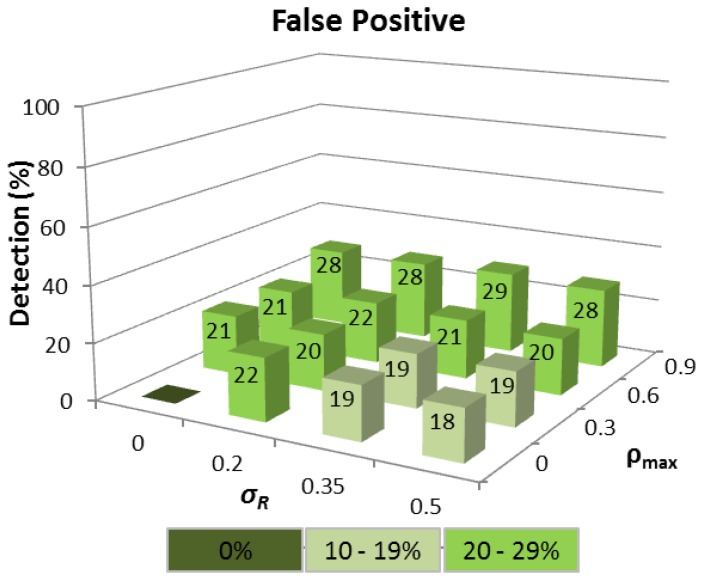
FP detection for different wormhole HC under variable network traffic loads (*ρ_max_*) and routing packet service times (*σ_R_*) with at least 15 Δ*T_i_* samples available.

The results reveal that for the ideal case where Δ*T_i_* is constant, so all nodes have identical hardware and multiprogramming level (*σ_R_= 0*), and each node carries negligible network traffic load (*ρ_max_= 0*), then 100% time tampering detection is achieved for all wormhole lengths with no corresponding FP being detected (see Figure 4). Predictably, as variations in Δ*T_i_* increase, the detection rate falls and FP increase, though the time tampering detection rate is still at least 86% for all wormhole lengths analysed even when *σ_R_*= 0.35 and *ρ_max_*= 0.6.

For wormhole lengths ≥5 hops, at least 94% of tampered Δ*T_i_* values can be successfully detected under all conditions when *σ_R_*= 0.5 and *ρ_max_*= 0.9, with the detection rate being 87% for a wormhole HC of 5. A notably aspect of the performance of the Δ*T Vector* extension, is that a minimum of 74% of tampered Δ*T_i_* values can still be detected even when the wormhole HC is 4. Pragmatically, this means that successful time tampering in wormholes ≥4 hops will be extremely difficult to achieve since the probability of avoiding detection is less than 30%.

For 3 HC wormholes, the time tampering detection performance drops markedly when there are variations in either network traffic load or routing packet service times, because a healthy node can then often produce a higher Δ*T_i_* than a tampered Δ*T_i_*. This reflects the situation of when heavy network traffic loads (*ρ ≈ 1)* unavoidably cause longer queuing delays and/or high multiprogramming levels lead to increased service times for routing packets. In contrast, the wormhole nodes and those nodes through which routing packets are tunneled may continue to have negligible loads (*ρ ≈ 0)* and correspondingly short packet service times.

Despite this decline in performance, tampered Δ*T_i_* values can still be detected with an accuracy of 57% for 3 HC wormholes, when *σ_R_*= 0.5 and *ρ_max_*= 0.9. This still characterises a noteworthy enhancement to TTHCA, especially when cognisance is made of the stringent criteria necessary to launch a time tampering attack in the first instance.

The corresponding FP detection rate remains ≈20% for the *σ_R_* range considered, provided *ρ_max_* ≤ 0.6 because the Q-test compares the difference between the two largest Δ*T_i_* values in relation to the difference between Δ*T_MAX_* and Δ*T_MIN_*, which will be approximately constant, regardless of the interval, provided the Δ*T_i_* values are linearly distributed. When *ρ_max_* = 0.9, the FP rate rises because the queuing delay of a node increases rapidly as *ρ* tends to 1, and the Δ*T_i_* distributions are no longer linear. This means that a Δ*T_i_* value produced by a node with a high network traffic load can easily become confused with a tampered Δ*T_i_*. Realistically however, even a FP rate of *≈*30% is still a satisfactory outcome since FP detection does not automatically mean that a route between a source and destination node cannot be established, but rather that an alternative route must be chosen other than the shortest path in terms of HC.

### 5.2. CASE 2: MANET Nodes without ΔT_i_ Track Records

**Figure 5.**
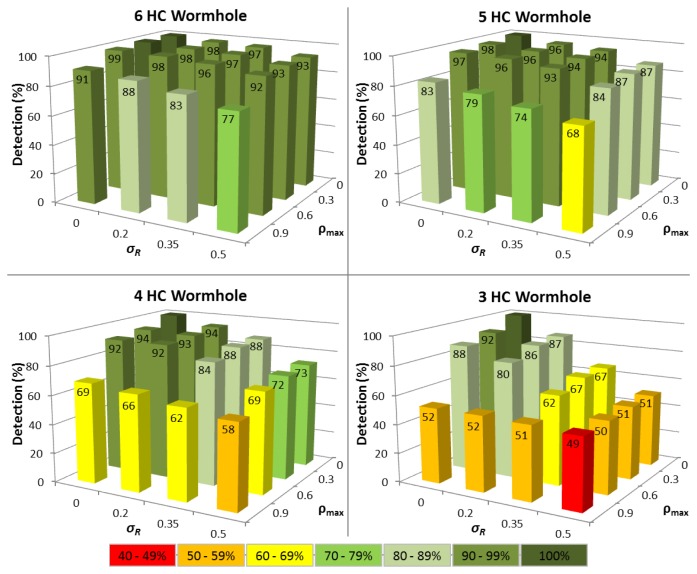
Time tampering detection performance for different wormhole HC under variable network traffic loads (*ρ_max_*) and routing packet service times (*σ_R_*) for 3 ≤ Δ*T_i_* samples ≤15.

The second set of experiments analysed the situation when a new node joins the MANET and requests a route for the first time. The same conditions are employed as in Section 5.1, though now it is assumed that only between three and 15 Δ*T_i_* values are available for the node requesting the new route, since there is no *a priori* knowledge about previously measured Δ*T_i_* values. The corresponding time tampering detection results are displayed in Figure 5.

**Figure 6.**
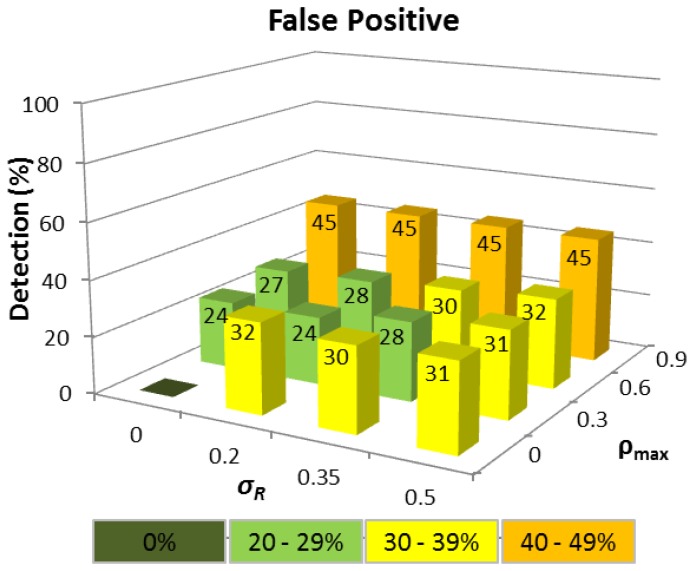
False positive detection for different wormhole HC under variable network traffic loads (*ρ_max_*) and routing packet service times (*σ_R_*) for 3 ≤ Δ*T_i_* samples ≤ 15.

The absence of any track record meant that detection performance was not as consistent as CASE 1, though a time tampering detection rate of ≥80% has still been achieved for all wormhole HC when *σ_R_* ≤ 0.2 and *ρ_max_* ≤ 0.6. For wormholes ≥5 hops, at least 68% of tampered Δ*T_i_* values were correctly detected even when *σ_R_*= 0.5 and *ρ_max_*= 0.9. The equivalent FP detection rates displayed in Figure 6, were slightly higher than in CASE 1 for *ρ_max_* ≤ 0.6 for example, and performance was more sensitive to high network traffic load variations (*ρ_max_* = 0.9) due to the smaller number of Δ*T_i_* samples. Nevertheless, even a FP rate of 45% when *ρ_max_*= 0.9 can still be deemed acceptable as more than half of all possible routes are available.

The time tampering detection performance is thus less robust in CASE 2 when no Δ*T_i_* track record is available, though this does represent the worst possible MANET situation, when a new node performs its first route discovery procedure. As a node runs the route discovery procedure more often, the corresponding time tampering detection rate will quickly improve and converge towards the results presented for CASE 1. This infers that to strengthen the time tampering detection performance for new nodes, it is prudent to run a few route discovery procedures before starting to communicate within the network. This could for instance, be accomplished by specifying within the routing protocol that a node is not allowed to communicate within the network until it has collected a minimum of 15 Δ*T_i_* samples.

### 5.3. Network Overheads and Computational Complexity

One of the consequences of the Δ*T Vector* extension is a larger RREP packet as it must contain the individual Δ*T_i_* values of all intermediate nodes of a route, while the original TTHCA mechanism only requires the sum Δ*T_TOT_*. The size of the Δ*T Vector* is dependent on the route HC, so if for example each Δ*T_i_* value is represented by 32 bits, then on a route from a source node S to a destination node D with intermediate nodes I_1_ and I_2_, RREP will comprise a Δ*T Vector* length of 32 bits, 64 bits and 96 bits when respectively received by I_2_, I_1_ and S. This contrasts with the corresponding RREP packet in the TTHCA algorithm which will have a 32 bits Δ*T_TOT_* value for each node. While a Δ*T Vector* with more than one element theoretically increases the transmission and reception time requirements for the routing packet, when cognisance is taken of the high bandwidths available in modern wireless technologies, the extended RREP packets will have negligible impact upon performance.

A second ramification of the Δ*T Vector* extension is the increased FP detection rate. From the network performance perspective, this means that the shortest route in terms of HC is not always available, as highlighted in both Sections 5.1 and 5.2. This does not necessarily imply decreased performance in terms of route delay since FP detection can in many cases lead to a positive outcome as routes with intermediate nodes with very high traffic loads will be omitted.

A formal complexity analysis for the new Δ*T Vector* extension reveals the only supplementary cost incurred compared with the original TTHCA algorithm is the outlier detection scheme performed by the source node. If the Dixon Q-test is used as the outlier method, the only extra computations needing to be performed relate to the ranking of Δ*T Vector* values. Since the Δ*T Vector* length equals the route HC, the time complexity for ranking is *O(*HC*^2^)*. This ranking however, can be implemented as a linear search of 4 Δ*T* values, since the Q-test only uses the three largest and the smallest Δ*T* value. This results in a time complexity for the new Δ*T Vector* extension of *O(*HC*)*, which is the same as TTCHA [17]. The corresponding FP performance of Δ*T Vector* extension also needs to be analysed because these are identified even when there are no errors in the measured node processing times. If the probability of a FP is *p*, then the probability of *i* FP occurring before a healthy route is located will be (*1−p*)*·pi*. The average number of route discovered before a healthy route can therefore be expressed as *p/*(*1−p*). So for *p < 0*.5, on average up to one FP will be discovered before a healthy route is identified for the Δ*T Vector* extension. The worst case in a single wormhole MANET is thus, on average three algorithm executions when a wormhole infected route is found before a healthy route is located. In contrast, the impact of FP on the TTHCA algorithm is less problematic because a FP is only identified when there are time measuring errors [17].

In summary, this formal analysis has shown the new Δ*T Vector* extension has the same linear time complexity as the original TTHCA algorithm, with the rider that because of FP occurrences, one additional execution cycle of the Δ*T Vector* extension may be necessitated, though this still affords a very effective lightweight protection mechanism against time tampering for TTHCA.

## 6. Conclusions and Future Research

The *traversal time and hop count analysis* (TTHCA) algorithm is a MANET wormhole detection technique, introduced as an extension to the *ad hoc distance vector* (AODV) routing protocol. A latent security threat to TTHCA is that as each intermediate node and the destination node measures the packet traversal time, a *participation mode* (PM) wormhole node can potentially provide false measurement values and avoid detection. This paper has analysed the conditions for a time tampering attack and proposed a security mechanism for TTHCA called the Δ*T Vector* extension for detecting false time values in PM *in-band* (I-B) wormholes. This requires the destination node and each intermediate node to add their individual processing times of the *route request* (RREQ) and *route reply* (RREP) packages (Δ*T_i_*) to a vector parameter in the RREP instead of using a single total packet processing time parameter (Δ*T_TOT_*) as in the original TTHCA algorithm. This makes each individual Δ*T_i_* measurement available for a node requesting a route and suspicious Δ*T_i_* values caused by PM I-B wormhole nodes can thus be identified by an outlier detection method. The Δ*T Vector* extension offers a notable security enhancement to the original TTHCA wormhole detection algorithm by providing an effective time tampering detection mechanism for PM wormholes, while retaining many of the smart features of TTHCA, particularly being a low-cost algorithm in terms of both computational complexity and network overheads.

In terms of future research, minimisation of *false positive* (FP) detections incurred by the Δ*T Vector* extension is an important objective. The FP rate can potentially be decreased by not including nodes suspected of time tampering to the *graylist*, since a high Δ*T_i_* caused by time tampering is permanent compared with a temporarily high Δ*T_i_* due to queuing delays. An alternative strategy is to choose a higher confidence level for the outlier detection, though this will proportionally reduce the corresponding time tampering detection performance of the Δ*T Vector* mechanism.

## Figures and Tables

**Table 1.** Simulation parameter settings.

**Parameter**	**Settings**

Distance between two successive nodes (*d*)	Randomly set: 150 m–250 m
Packet propagation speed (*S*)	3 × 10^8^ m/s
Routing packet service time per node distribution (*T_S_*)	Randomly chosen from linear probability distributions for variable *σ_R_*
Routing packet processing time per node distribution (Δ*T_RREQ/RREP_*)	Calculated from Equation (11)
Network traffic load per node distribution (*ρ*)	Randomly *0* ≤ *ρ* ≤ *ρ_max_* for variable *ρ_max_*
Route HC	Randomly set: 3–15
Number of samples per test case	100,000
Wormhole attack type	PM I-B
Time tampering attack	Launched according to Equations (7) and (8)

## Appendix A

### Custom Tool for Simulating Different Sized Δ*T Vectors*

This section investigates the software tool used to generate the simulated Δ*T_i_* values, with all variables with their initialised values being displayed in Table A1. To enable the interested reader to faithfully reproduce this tool, the documented pseudo-code showing the creation of a Δ*T_i_Vector* during both the RREQ broadcast and corresponding RREP response phases is provided in Tables A2 and A3 respectively.

**Table A1.** Variables used in the simulation tool and their initial values.

**Variable**	**Description**
*HC_WH_* = user defined	Wormhole length (number of hops)
*HC* = [3,15]	Randomly chosen for each route. Includes both the actual route *HC* and *HC_WH_*
*t_RREQ_* = *0*	Tunnelling delay of RREQ through wormhole link
*t_RREP_*= = *0*	Tunnelling delay of RREP through wormhole link
*M_1_* = *1*	Malicious node #1
*M_2_* = *M_1_*+ *HC_WH_*	Malicious node #2
*i* = *1*	Δ*T Vector* index

**Table A2.** Routing packet processing delay generation and malicious node time tampering estimations during the RREQ broadcast phase.

**Code Section**	**Description/motivation**
FOR *I* = 1 to *HC*:	*I=1* is the first intermediate node and *I* = *HC* is the destination node
*ρ* = [ *0*, *ρ_max_*]	Random traffic load assigned for each node
*T_S-I_* = randomly chosen from a linear distribution with user defined relative standard deviation (*σ_R_*)	Every node (*I*) is assigned a random packet service time.
Δ*T_RREQ-I_* calculated according to Equation (11)	RREQ processing time at each node calculated according to the M/D/1 queuing model.
*d_I_*= random between 150m and 250m	Distance between node *I* and *I-1*
IF *I* > *M_1_* AND *I* < *M_2_* THEN *t_RREQ_* = *t_RREQ_* + *d_I_/S* + Δ*T_RREQ-I_*	RREQ tunnelled propagation delay through the wormhole is the sum of Δ*T_RREQ-I_* at each intermediate node and PTT between *M_1_* and *M_2_*.
IF *I* = *M_2_* THEN *t_RREQ_* = *t_RREQ_* + *d_I_/S* Δ*T_F2_* = *t_RREQ_* + *R/S*	PTT between *I (M_2_)* and *I-1* is added to *t_RREQ_* and M_2_ calculates Δ*T_F1_* to be added to its Δ*T_i_* when it receives the corresponding RREP.
END FOR	

**Table A3.** Routing packet processing delay generation, time tampering and generation of the Δ*T Vector* during the RREP response phase.

**Code Section**	**Comments**
FOR *I* = HC to *1*:	As it is a RREP broadcast, the iteration starts at *I* = *HC*.
*ρ *= [ *0*, *ρ_max_*]	Random traffic load is assigned for each node to reflect a potential change in network traffic conditions between processing RREQ and RREP.
Δ*T_RREP-I_* calculated according to Equation (11)	RREP processing time at each *I* follows the M/D/1 queuing model. Since the service time of both RREQ and RREP is assumed constant, *T_S-I_* from Table A2 is used.
Δ*T_i_* = Δ*T_RREQ-I_* + Δ*T_RREP-I_*	Processing delays of both RREQ and RREP added to the Δ*T Vector* (Δ*T_i_*)
IF *I* < *M_2_* AND *I* > *M_1_* THEN *t_RREP_* = *t_RREP_* + *d_I_/S* + Δ*T_RREP-I_*	RREP tunnelled propagation delay through the wormhole is the sum of all Δ*T_RREP_* at intermediate nodes and the PTT between *M_2_* and *M_1_*.
ELSE Increment *i*	If *I* is a legitimate intermediate node then Δ*T Vector* index is incremented.
IF *I* = *M_2_* THEN *t_RREP_* = *t_RREP_* + *d_i_/S* Δ*T_i_* = Δ*T_i_* + Δ*T_F2_*	The PTT between *I (M_2_)* and *I-1* is added to *t_RREP_* and *M_2_* increments its entry in Δ*T Vector* with Δ*T_F2_* which was calculated during the RREQ broadcast process.
IF *I* = *M_1_* THEN Δ*T_F1_* = *t_RREP_* + *R/S* Δ*T_i_* = Δ*T_i_* + Δ*T_F1_*	*M_1_* calculates Δ*T_F1_* with which Δ*T_i_* is incremented.
END FOR	
